# Intermittent energy restriction and risk of physician-diagnosed diabetes progression: a propensity-weighted real-world cohort study

**DOI:** 10.3389/fnut.2026.1744017

**Published:** 2026-02-04

**Authors:** Zhiyong Xiao, Xu Zhou, Dongliang Yang, Xihu Lai, Yewu Zhang, Ruiyu Wu, Huiqing Wang, Jiali Zhou, Xiao Yang, Feng Xu, Wu Luo, Xuan Chen, Bin Zhou, Xinhong Yin, Dongbo Liu

**Affiliations:** 1 Horticulture College, Hunan Agricultural University, Changsha, China; 2 School of Nursing, University of South China, Hengyang, China; 3 Yuelushan Laboratory, Changsha, China; 4 Department of General Education Courses, Cangzhou Medical College, Cangzhou, China; 5 Xincheng Smart Internet Hospital, Chengdu, China; 6 Chinese Center for Disease Control and Prevention, Beijing, China; 7 Hunan Provincial Engineering Research Centre of Medical Nutrition Intervention Technology for Metabolic Diseases, Changsha, China; 8 Hepatobiliary and Pancreatic Surgery Department, First Hospital of University of South China, Hengyang, China; 9 Three Gorges University Hospital of Traditional Chinese Medicine & Yichang Hospital of Traditional Chinese Medicine, Yichang, China; 10 Department of Endocrinology, The Central Hospital of Shaoyang, Shaoyang, Hunan, China

**Keywords:** glycemic control, intermittent energy-restricted, physician-diagnosed diabetes progression, real-world evidence, retrospective cohort study

## Abstract

**Background:**

Intermittent energy restriction (IER) shows metabolic promise, but real-world evidence for its impact on clinically meaningful outcomes is lacking. This study evaluated the effect of IER on a novel composite endpoint of early, physician-diagnosed diabetes progression (PDDP), which includes microvascular complications (retinopathy, nephropathy, neuropathy), acute metabolic decompensation (diabetic ketoacidosis, hyperosmolar hyperglycemic state), and peripheral arterial disease.

**Materials and methods:**

In a large real-world retrospective cohort study, 1,069 participants following a structured 5:10 IER regimen were compared with 1,099 controls. The primary outcome was the incidence of PDDP, a composite of the progression of diabetes-related events diagnosed by clinical doctors. Secondary outcomes included fasting plasma glucose (FPG) change and diabetes medication reduction. Inverse probability weighting (IPW) and multivariate models were used to control for confounders. A sensitivity analysis was restricted to patients without PDDP at baseline (*n* = 1,788).

**Results:**

The IER group demonstrated a substantially lower incidence of PDDP compared to controls (2% vs. 10%; *p* < 0.001). After IPW adjustment, the IER cohort demonstrated a significantly lower incidence of PDDP (Estimate: −0.09; 95% CI: −0.12 to −0.07; *p* < 0.001), and a greater reduction in antidiabetic medication use (OR: 6.26; 95% CI: 5.61 to 6.99; *p* < 0.001), no statistically significant difference in FPG change was detected between groups (Estimate: −0.07; 95% CI: −0.46 to 0.32; *p* = 0.740). Sensitivity analysis confirmed the robustness of these benefits in the population without PDDP at baseline.

**Conclusion:**

In a real-world setting, IER was associated with a dramatically lower risk of PDDP, independent of its glucose-lowering effect. These findings position IER as a potent, scalable non-pharmacological strategy to improve patient-centered outcomes in diabetes care.

## Introduction

The worldwide prevalence of type 2 diabetes is increasing, underscoring the critical necessity for effective strategies that address not only hyperglycemia but, more importantly, its debilitating complications (1), which collectively contribute to 60% of global diabetes-related mortality (2). While pharmacological advancements, including glucagon-like peptide-1 (GLP-1) receptor agonists and sodium-glucose cotransporter 2 (SGLT2) inhibitors, have improved glycemic outcomes (3, 4), their ability to consistently reduce complications remains uncertain, and accessibility especially in low-resource settings is severely limited (5, 6). Furthermore, foundational trials such as ACCORD revealed a sobering truth: intensive glucose control alone may be insufficient to mitigate cardiovascular risk (7), exposing a fundamental gap in our therapeutic paradigm.

While randomized trials have established the efficacy of various interventions for glycemic control, their power to detect differences in long-term classic complications is limited by the need for extended follow-up periods, often exceeding a decade (8). This creates a critical translational gap: clinicians and patients require evidence on whether an intervention halts disease progression within a meaningful, shorter timeframe. There is a growing recognition that earlier, clinically apparent milestones of diabetes progression that are actionable in real-time clinical practice may serve as robust surrogate endpoints that are both patient-important and feasibly measurable within real-world studies (9).

Intermittent energy restriction (IER) has emerged as a promising non-pharmacological strategy (10, 11), mechanistic studies suggest that regimens incorporating periodic energy restriction can improve insulin sensitivity, reduce oxidative stress, and promote metabolic adaptation (12, 13). Evidence suggests IER can halt the diabetes progression such as Diabetic Kidney Disease (DKD) (14), and achieve comprehensive cardiometabolic health optimization (15). However, a significant evidence gap persists: whether these metabolic benefits translate into a reduction in tangible, real-world clinical events. Existing studies are hampered by a reliance on long-term complications—endpoints that require decades to observe—thus failing to provide timely evidence for clinical decision-making.

To address this evidence gap, we conducted a large scale, real world cohort study to evaluate the effect of IER on a novel (16), clinically grounded composite outcome termed Physician Diagnosed Diabetes Progression (PDDP) which is introduced for the first time in this study. This endpoint captures early, meaningful clinical events that reflect actual disease advancement and are readily identifiable in routine practice. The primary objective of this study is to determine whether IER reduces the risk of diabetes progression independent of its glucose lowering effects. By focusing on outcomes that truly matter to patients and are feasible to assess within a pragmatic timeframe, this work aligns with the WHO Global Diabetes Compact (17), and provides timely, actionable evidence to guide clinical decision making and health policy.

## Methods

### Study design and data sources

The retrospective study was conducted as part of the CMNT programme (16) period extended from January 1, 2022 to July 31, 2023, with follow-up ending on January 31, 2024. The longest duration was analyzed for patients with multiple follow-up visits to evaluate the intervention’s effects comprehensively. Given the real-world context of the intervention, randomization was impractical; therefore, inverse probability weighting (IPW) was employed to address baseline differences between the IER and control cohorts have been comprehensively reported in our prior work (16). The inclusion criteria for both cohorts were as follows: age 18 to 75 years, body mass index (BMI) between 18 and 35 kg/m^2^, and a confirmed diagnosis of type 2 diabetes. Patients with a follow-up period shorter than 3 months were excluded. A minimum follow-up duration of 3 months was required to ensure sufficient data collection for outcome evaluation.

IER cohort followed a structured 5:10 intermittent fasting regimen, comprising six 15-day cycles. Each cycle included 5 days of strict calorie restriction (917 kcal/day) (Supplementary Table S1) followed by 10 days of usual diet, guided by the Dietary Guidelines for Diabetes in China (2017 Edition). Adherence was supervised by health managers via a digital delivery model using videoconferencing, with compliance monitored through daily dietary logs. Medication management was overseen by physicians, who adjusted or discontinued antidiabetic medications according to established RCT protocols to ensure patient safety and adherence. Given the real-world, retrospective design of this study, participants were included in the IER cohort only if they completed the prescribed regimen under supervision; thus, this cohort represents a per-protocol population for assessing the effectiveness of the intervention.

The control cohort was selected from outpatient registries of the First Affiliated Hospital of the University of South China. Eligibility criteria matched those of the IER cohort: age 18–75 years, BMI 18–35 kg/m^2^, type 2 diabetes diagnosis, and follow-up ≥3 months. Control participants received usual care following the Dietary Guidelines for Diabetes in China (2017 Edition), including routine dietary counseling, pharmacotherapy, and metabolic monitoring. To minimize selection bias, controls were enrolled consecutively during the same study period (January 2022–July 2023) and were not participating in any structured dietary intervention program.

Data collected from hospital database included demographic and clinical characteristics such as gender, age, BMI, diabetes duration, FPG, medication use, incidence of PDDP. Baseline and follow-up clinical assessments were conducted using standardized protocols to ensure consistency. Missing data accounted for less than 1% of the dataset. Multiple imputation was employed for sporadically missing variables by using medians for continuous variables and modes for categorical variables. For primary outcomes (PDDP incidence), no imputation was performed; events were based on complete physician-diagnosed records.

This study complied with the Strengthening the Reporting of Observational Studies in Epidemiology (STROBE) guidelines. Ethical approval was granted by the Medical Ethics Committee of the University of South China (Approval No. 2023NHHL090).

### Outcomes

The primary outcome was the incidence of physician-diagnosed diabetes progression (PDDP), defined as the first occurrence of any component of a clinically defined composite endpoint during the follow-up period. PDDP components included: (1) Microvascular disease progression, comprising diabetic retinopathy (ICD-10: E11.3, H36.0) diagnosed via fundus photography or ophthalmologist assessment; diabetic nephropathy (ICD-10: E11.2, N08.3) defined as new-onset albuminuria (UACR ≥30 mg/g) or an eGFR decline >5 mL/min/1.73m^2^/year; and diabetic neuropathy (ICD-10: E11.4, G63.2) diagnosed based on clinical symptoms and confirmatory nerve conduction studies. (2) Acute metabolic decompensation, including diabetic ketoacidosis (DKA; ICD-10: E11.1) or hyperosmolar hyperglycemic state (HHS; ICD-10: E11.0) requiring hospitalization. (3) Macrovascular and other complications, such as peripheral arterial disease (ICD-10: E11.5, I73.9) diagnosed via an ankle-brachial index <0.9 or vascular imaging, diabetic foot (ICD-10: E11.6), or incident cardiovascular events (ICD-10: I21-I25). All events were physician-diagnosed and extracted from structured electronic health records using standardized ICD-10 codes. Event adjudication was performed by two independent clinicians, with discrepancies resolved by a third senior endocrinologist. PDDP was considered present if any component event was newly recorded after baseline and confirmed by clinical documentation. Secondary outcomes comprised: (1) FPG change, calculated as the difference between baseline and follow-up values; (2) reduction in diabetes medication use, defined as a decrease in the number of medications between baseline and follow-up. The cohort data and analyses pertaining to certain secondary efficacy indicators (medication reduction and fasting blood glucose change) presented in this study have been utilized in prior research examining the metabolic effects of IER on type 2 diabetes remission (16). However, the present manuscript addresses a distinct and novel scientific question, focusing explicitly on the progression of diabetes as defined by the novel composite endpoint of Physician-Diagnosed Diabetes Progression (PDDP).

### Statistical analysis

Univariate analyses were conducted using the CBCgrps package in R. Continuous variables with normal distributions are presented as mean ± standard deviation, while those with non-normal distributions are presented as median [Q1, Q3]. Categorical variables are described using frequency (*n*) and percentage (%). Differences in baseline characteristics were evaluated using independent samples *t*-tests, Mann–Whitney U tests, and chi-square tests as appropriate.

Multivariate analyses utilized Generalized Linear Mixed-Effects Models (GLMM) for binary outcomes and Linear Mixed-Effects Models (LMM) for continuous outcomes, implemented using the lme4 package in R. GLMMs accounted for both fixed effects (e.g., treatment group, clinical variables) and random effects (e.g., region), adjusting for geographic variations and patient population differences. Covariates included treatment group, follow-up duration, baseline FPG, PDDP, insulin use, age, and medication count. Inverse Probability Weighting (IPW) was applied using the PS weight package in R to balance baseline covariates between cohorts (16), enhancing comparability and supporting valid causal inferences regarding intervention effects. Following IPW balancing, Cox regression and linear regression models evaluated the relationships between the intervention and various clinical outcomes, including incidence of PDDP, medication reduction and changes in FPG.

Subgroup analyses employed propensity score-weighted Cox proportional hazards regression models to assess hazard ratios (HR) within specific patient subgroups, validating the consistency and robustness of intervention effects. Subgroups were delineated based on insulin use (users vs. non-users), diabetes duration (>5 years vs. ≤5 years), BMI (<24 kg/m^2^ vs. ≥24 kg/m^2^), medication use (multiple vs. fewer medications), and follow-up FPG levels (<7 mmol/L vs. ≥7 mmol/L) which is widely regarded as an optimal control target for patients with diabetes (18).

For the primary analysis, patients with existing PDDP at baseline were included, and baseline PDDP status was treated as a key covariate for adjustment to control for differential baseline risk. To specifically assess the effect on new-onset progression and address potential bias, a pre-specified sensitivity analysis was performed excluding all patients with any PDDP component at baseline (*n* = 1,788). In the sensitivity analysis, we restricted the study population to patients without complications at baseline to assess the robustness of the intervention (ICR) effect on primary outcomes. Post-IPW covariate balance was assessed using standardized mean differences (SMD), with SMD < 0.1 considered well-balanced, as detailed in Supplementary Figure S1.

All statistical analyses were conducted using R software (version 4.2.3), with statistical significance set at *α* = 0.05. This comprehensive approach, leveraging advanced models and weighting techniques, ensures that findings are rigorous and applicable in real-world settings.

## Results

A total of 1,069 participants in the IER group and 1,099 in the control group were analyzed from January 1, 2022 to July 31, 2023. Baseline characteristics differed significantly between cohorts: the IER group was younger (median age 55 versus 60 years; *p* < 0.001), exhibited lower baseline FPG (7.9 versus 9.1 mmol/L; *p* < 0.001), and included fewer insulin users (15% versus 41%; *p* < 0.001) (Table 1). Adjustments for baseline PDDP (Supplementary Table S3), age, baseline FPG, antidiabetic medication use (Supplementary Table S2), follow-up durations, and insulin use were performed via IPW as described before (16).

**Table 1:** Baseline characteristics of IER and control cohorts.

Variables	Total (*n* = 2,168)	IER (*n* = 1,069)	Control (*n* = 1,099)	*p*-value
Sex				0.093
Male	1,358 (63)	689 (64)	669 (61)	
Female	810 (37)	380 (36)	430 (39)	
Follow-up duration (days)	171.94 (124.28, 242)	152.21 (119.89, 200.03)	200 (135, 304.5)	<0.001
FPG at baseline (mmol/L)	8.6 (7.1, 11.4)	7.9 (6.6, 9.7)	9.12 (7.98, 13.61)	<0.001
PDDP at baseline				<0.001
0	1788 (82)	1,069 (100)	719 (65)	
1	361 (17)	0 (0)	361 (33)	
2	19 (1)	0 (0)	19 (2)	
Insulin use at baseline				<0.001
Untreated	1,561 (72)	913 (85)	648 (59)	
Treated	607 (28)	156 (15)	451 (41)	
Age (years)	58 (51, 64)	55 (50, 60)	60 (54, 68)	<0.001
BMI (kg/m^2^)	23.64 (21.66, 25.59)	23.53 (21.8, 25.35)	23.81 (21.48, 25.93)	0.486
Antidiabetic drugs at baseline				<0.001
0	313 (14)	245 (23)	68 (6)	
1	906 (42)	367 (34)	539 (49)	
2	680 (31)	335 (31)	345 (31)	
3	217 (10)	98 (9)	119 (11)	
4	52 (2)	24 (2)	28 (3)	
Duration of diabetes (years)	5 (2, 10)	5 (2, 10)	6 (1, 10)	0.112

This table presents baseline demographic and clinical characteristics of IER and control cohorts, including age, FPG, BMI, duration of diabetes, insulin use, and antidiabetic medication use. Statistically significant differences between cohorts are indicated by *p* < 0.05.

Univariate analysis revealed the incidence of PDDP was significantly lower in the IER cohort compared to controls (*p* < 0.001). Specifically, 98% (1,050/1,069) of IER patients developed no PDDP, versus 90% (988/1,099) in the control group. Furthermore, drug reduction was achieved in a significantly higher proportion of IER patients (61%, 651/1,069) compared to controls (22%, 237/1,099; *p* < 0.001). In contrast, the median change in FPG was identical (−1.4) in both cohorts, with no statistically significant difference between groups (*p* = 0.346) (Table 2).

**Table 2:** Univariate analysis of outcomes for IER and control cohorts.

Variables	Total (*n* = 2,168)	IER (*n* = 1,069)	Control (*n* = 1,099)	*p*-value
New-onset PDDP				<0.001
0	2038 (94)	1,050 (98)	988 (90)	
1	105 (5)	13 (1)	92 (8)	
2	25 (1)	6 (1)	19 (2)	
FPG change	−1.4 (−3.2, 0.1)	−1.4 (−2.9, −0.1)	−1.4 (−3.72, 0.86)	0.346
Drugs reduction				<0.001
Reduce	888 (41)	651 (61)	237 (22)	
No reduction	1,280 (59)	418 (39)	862 (78)	

Univariate analysis comparing FPG changes, incidence of new-onset complications, and drugs reduction between IER and control cohorts. *p*-values below 0.05 indicate statistically significant differences between the cohorts.

Multivariate regression analyses, adjusted for key clinical covariates, demonstrated that intermittent energy restriction (IER) was a statistically significant independent predictor for all three primary outcomes. Patients in the IER group exhibited a substantially lower risk of new-onset PDDP compared to controls (IRR = 0.34, 95% CI: 0.20–0.53, *p* < 0.001). Conversely, IER was associated with significantly greater reduction in FPG (OR = 4.2, 95% CI: 2.39–7.40, *p* < 0.001) and a higher likelihood of antidiabetic drug reduction (Estimate = −1.45, 95% CI: −1.84 to −1.05, *p* < 0.001). Among other covariates, baseline insulin use was significantly associated with increased incidence of PDDP (IRR = 2.31, 95% CI: 1.64–3.26, *p* < 0.001) but with reduced FPG change (OR = 0.2, 95% CI: 0.08–0.52, *p* = 0.001). Higher baseline FPG was predictive of greater FPG reduction (OR = 0.83, 95% CI: 0.77–0.89, *p* < 0.001) and drug reduction (Estimate = −0.7, 95% CI: −0.74 to −0.66, *p* < 0.001). Follow-up duration and age showed modest but significant associations with selected outcomes (Table 3).

**Table 3:** Multivariate analysis of outcome indicators.

Predictors	Incidence of PDDP		FPG change		Drugs reduction	
	IRR (95% CI)	*p*-value	OR (95% CI)	*p*-value	Estimates (95% CI)	*p*-value
IER	0.34 (0.20–0.53)	<0.001	4.2 (2.39–7.40)	<0.001	−1.45 (−1.84 to −1.05)	<0.001
Follow-up years	1.0007 (0.9995–1.0020)	0.252	0.99 (0.99–0.99)	<0.001	0.00085 (−0.0006 to 0.0023)	0.262
FPG at baseline	0.97 (0.93–1.00)	0.095	0.83 (0.77–0.89)	<0.001	−0.7 (−0.74 to −0.66)	<0.001
PDDP at baseline	1.504 (1.10–2.05)	0.01	1.18 (0.44–3.11)	0.743	0.25 (−0.18 to 0.68)	0.253
Insulin use at baseline	2.31 (1.64–3.26)	<0.001	0.2 (0.08–0.52)	0.001	1.18 (0.81–1.56)	<0.001
Age	1.01 (0.99–1.03)	0.24	0.98 (0.95–1.0)	0.035	0.02 (0.00–0.04)	0.041
Antidiabetic drugs at baseline	1.22 (1.03–1.45)	0.023	0.27 (0.22–0.35)	<0.001	0.003 (−0.17 to 0.17)	0.969

Multivariate analysis of drug reduction, incidence of PDDP, and FPG changes between the IER and control cohorts, adjusted for age, baseline FPG, and insulin use. Odds ratios (OR) and 95% confidence intervals (CI) are presented. Significant predictors are indicated with *p* < 0.05. Covariates included treatment group, follow-up duration, baseline FPG, baseline PDDP, insulin use, age, and medication.

Based on the unadjusted incidence data (Supplementary Table S3), the absolute risk of PDDP was 1.78% in the IER cohort and 9.92% in the control cohort, yielding an absolute risk reduction (ARR) of 8.14%. The corresponding number needed to treat (NNT) to prevent one PDDP event was 12. Following IPW adjustment and quantitatively demonstrates the standardized mean differences (SMD) for all key covariates before and after IPW weighting, confirming that excellent balance (SMD < 0.1) was achieved, the IER was associated with a statistically significant reduction in PDDP (Estimate = −0.09, 95% CI: −0.12 to −0.07, *p* < 0.001). Similarly, a strong positive effect was observed for drug reduction outcomes (Estimate = 6.26, 95% CI: 5.61–6.99, *p* < 0.001). In contrast, the analysis did not demonstrate a significant effect for FPG change (Estimate = −0.07, 95% CI: −0.46 to 0.32, *p* = 0.740) (Table 4).

**Table 4:** Outcomes after IPW.

Variables	Estimate (95% CI)	Std. error	*p*-value
Incidence of PDDP (0, 1, 2)	−0.09 (−0.12 to −0.07)	0.01	<0.001
FPG change	−0.07 (−0.46 to 0.32)	0.20	0.740
Drugs reduction	6.26 (5.61–6.99)	0.35	<0.001

This table presents reduction in medication use, changes in FPG, and incidence of new-onset complications after adjustment for confounders using IPW. Estimates and 95% CIs are provided, with statistical significance indicated by *p* < 0.05.

Subgroup analyses using inverse probability weighting revealed consistent beneficial effects of intermittent energy restriction (IER) across most patient populations, with significant treatment effect heterogeneity observed in key subgroups. For the primary outcome of new-onset PDDP, IER demonstrated superior protective effects compared to controls across all subgroups (all *p* < 0.05), except in patients without antidiabetic drugs at baseline (*p* = 0.724). Significant treatment effect modification was observed for BMI (*p*-interaction = 0.003), diabetes duration (*p*-interaction < 0.001), baseline antidiabetic drug use (*p*-interaction < 0.001), and insulin treatment status (*p*-interaction = 0.004). Notably, the protective effect was more pronounced in patients with lower BMI (IRR = 0.209) and shorter diabetes duration (IRR = 0.582) (Table 5).

**Table 5:** Subgroup analysis of outcomes after IPW for IER and control cohorts.

Outcome	IER Crude incidence rate (95% CI)	Control Crude incidence rate (95% CI)	IER versus control Estimate (95% CI)	*p*-value	*p*-interaction
Incidence of new-onset PDDP					
BMI ≥ 24	0.059 (0.056–0.061)	0.157 (0.155–0.159)	0.352 (0.2030–0.5746)	<0.001	
BMI < 24	0.045 (0.044–0.047)	0.205 (0.203–0.208)	0.209 (0.1289–0.3225)	<0.001	0.003
PDDP of diabetes at Baseline = 0	0.051 (0.050–0.052)	0.130 (0.129–0.132)	0.317 (0.219–0.447)	<0.001	
Duration of diabetes ≥ 5	0.050 (0.048–0.052)	0.271 (0.268–0.274)	0.153 (0.0932–0.2362)	<0.001	
Duration of diabetes < 5	0.051 (0.050–0.053)	0.090 (0.088–0.091)	0.582 (0.3347–0.9685)	0.044	<0.001
With antidiabetic drugs	0.058 (0.057–0.059)	0.189 (0.188–0.190)	0.270 (0.1865–0.3798)	<0.001	
Without antidiabetic drugs	0.021 (0.018–0.024)	0.073 (0.060–0.0865)	0.752 (0.1443–4.0146)	0.724	<0.001
FPG follow-up ≥ 7	0.067 (0.064–0.070)	0.204 (0.203–0.206)	0.294 (0.1772–0.4595)	<0.001	
FPG follow-up < 7	0.042 (0.040–0.043)	0.122 (0.118–0.125)	0.247 (0.1452–0.4035)	<0.001	0.098
With insulin treatment	0.061 (0.055–0.067)	0.299 (0.295–0.302)	0.151 (0.0745–0.2717)	<0.001	
Without insulin treatment	0.049 (0.048–0.050)	0.101 (0.099–0.102)	0.484 (0.3102–0.7365)	0.001	0.004
FPG change					
BMI ≥ 24	−1.787 (−2.162 to −1.411)	−1.596 (−2.197 to −0.996)	−1.246 (−1.834 to −0.658)	<0.001	0.246
BMI < 24	−1.616 (−1.936 to −1.296)	−1.962 (−2.621 to −1.303)	−1.723 (−2.268 to −1.178)	<0.001	
PDDP of diabetes at Baseline = 0	−1.687 (−1.930 to −1.444)	−1.282 (−2.075 to −0.489)	−1.635 (−2.045 to −1.224)	<0.001	
Duration of diabetes ≥ 5	−1.790 (−2.156 to −1.425)	−0.470 (−1.109 to 0.169)	−3.019 (−3.626 to −2.411)	<0.001	<0.001
Duration of diabetes < 5	−1.604 (−1.93 to −1.278)	−3.166 (−3.759 to −2.572)	−0.410 (−0.927 to 0.108)	0.121	
With antidiabetic drugs	−1.545 (−1.820 to −1.270)	−1.716 (−2.180 to −1.252)	−1.530 (−1.983 to −1.076)	<0.001	0.671
Without antidiabetic drugs	−2.290 (−2.79 to −1.790)	−2.956 (−4.589 to −1.323)	−1.280 (−2.071 to −0.490)	0.002	
FPG follow-up ≥ 7	−0.953 (−1.418 to −0.488)	−0.461 (−0.961 to 0.039)	−3.050 (−3.680 to −2.421)	<0.001	<0.001
FPG follow-up < 7	−2.112 (−2.375 to −1.848)	−5.433 (−6.184 to −4.682)	1.395 (0.946 to 1.845)	<0.001	
With insulin treatment	−1.138 (−1.745 to −0.532)	−0.871 (−1.645 to −0.098)	−1.091 (−1.99 to −0.190)	0.018	0.319
Without insulin treatment	−1.796 (−2.061 to −1.532)	−2.430 (−2.956 to −1.902)	−1.580 (−2.024 to −1.136)	<0.001	
Drugs reduction					
BMI ≥ 24	1.38 (1.22–1.54)	0.31 (0.25–0.37)	3.27 (2.80–3.82)	<0.001	0.546
BMI < 24	1.29 (1.16–1.42)	0.36 (0.30–0.42)	2.78 (2.41–3.22)	<0.001	
PDDP of diabetes at Baseline = 0	1.33 (1.22–1.43)	0.32 (0.27–0.38)	2.78 (2.49–3.12)	<0.001	
Duration of diabetes ≥ 5	1.39 (1.23–1.54)	0.38 (0.31–0.44)	3.01 (2.58–3.50)	<0.001	0.99
Duration of diabetes < 5	1.28 (1.14–1.41)	0.29 (0.24–0.35)	3.01 (2.60–3.49)	<0.001	
With antidiabetic drugs	1.63 (1.51–1.76)	0.36 (0.31–0.40)	3.26 (2.93–3.62)	<0.001	NA
Without antidiabetic drugs	NA	NA	NA	NA	
FPG follow-up ≥ 7	1.23 (1.07–1.39)	0.31 (0.27–0.36)	2.94 (2.54–3.41)	<0.001	0.686
FPG follow-up < 7	1.38 (1.25–1.51)	0.39 (0.30–0.48)	2.61 (2.22–3.06)	<0.001	
With insulin treatment	1.69 (1.41–1.98)	0.39 (0.32–0.47)	3.52 (2.91–4.26)	<0.001	0.449
Without insulin treatment	1.25 (1.14–1.36)	0.29 (0.24–0.35)	2.88 (2.53–3.27)	<0.001	

Subgroup analyses of clinical outcomes after IPW in the IER and control cohorts. The table presents crude incidence rates and adjusted estimates with 95% CI for new-onset PDDP, FPG changes, and medication reduction across subgroups stratified by BMI, duration of diabetes, baseline PDDP, antidiabetic drug use, insulin treatment, and FPG follow-up levels. *p*-interaction indicates heterogeneity in treatment effects between subgroups. Data are presented as incidence rates (95% CI) or OR (95% CI), with statistically significant results (*p* < 0.05) highlighted.

Sensitivity analysis restricted to patients without baseline PDDP (*n* = 1,788) demonstrated findings consistent with the primary analysis. Baseline characteristics showed significant differences between IER and control cohorts in age, follow-up duration, baseline FPG, insulin use, and antidiabetic drug utilization (all *p* < 0.05), while sex, origin, and BMI were balanced (Supplementary Table S4-1). After inverse probability weighting adjustment (Supplementary Figure S1), IER remained strongly associated with reduced PDDP incidence (Estimate = 0.05, 95% CI: 0.04–0.08, *p* < 0.001) and increased drug reduction (OR = 5.34, 95% CI: 4.76–6.00, *p* < 0.001). The association with FPG change was not statistically significant (Estimate = 0.37, 95% CI: −0.05 to 0.79, *p* = 0.081) (Supplementary Table S4-3). The primary analysis included all eligible participants, with baseline PDDP status adjusted for as a covariate. In a pre-specified sensitivity analysis designed to assess incident progression, we restricted the cohort to patients without any PDDP events at baseline (*n* = 1,788). The consistency of findings across both analytical approaches supports the robustness of the observed association.

## Discussion

The primary outcome was the first occurrence of any component of a novel composite endpoint, termed Physician-Diagnosed Diabetes Progression (PDDP). This endpoint was specifically constructed for this study to capture early, clinically significant, and objectively verifiable milestones of diabetes advancement that are both actionable in routine practice and feasibly ascertainable within a real-world study time frame (19, 20). the use of a composite outcome of clinically apparent events aligns with the call for a greater focus on patient-important outcomes in diabetes research (9, 21).

This large-scale observational study provides evidence that IER is associated with a substantially lower risk of early diabetes progression. The primary innovation lies in the endpoint—Physician-Diagnosed Diabetes Progression (PDDP)—which captures actionable, early signs of microvascular and acute metabolic deterioration routinely identified in clinical practice (22). Our finding that IER was associated with a substantial reduction in PDDP risk (adjusted risk difference: −0.09) within a median follow-up of 5 months, potent effect on stabilizing metabolic health and altering the disease trajectory (23), shifting the therapeutic paradigm from preventing distant outcomes to arresting proximate progression. The novelty lies in their aggregation into the composite PDDP endpoint for use in real-world, shorter-term studies. This approach is justified by its high clinical face validity, feasibility of ascertainment within routine practice, and alignment with the need for patient-important outcomes measurable in pragmatic research timeframes. While PDDP demonstrates utility in this study, we present it as a construct that requires further prospective validation and refinement in independent cohorts.

The magnitude of risk reduction observed with IER appears to surpass what might be expected from glycemic improvement alone. The observed variations in FPG outcomes across analytical stages—from comparable unadjusted changes to a non-significant between-group difference after rigorous IPW adjustment—reflect the sequential control for baseline confounding. The univariate analysis presents the raw observed difference, while the IPW analysis provides the best estimate of the causal effect in a balanced population. Notably, the dissociation between the substantial reduction in PDDP risk and the attenuated FPG effect after balancing suggests that the benefits of IER on diabetes progression may extend beyond acute glycemic reduction. The progression of FPG results from unadjusted to IPW-adjusted analyses highlights the critical role of confounding control. The fact that IER’s robust protection against PDDP persisted despite an attenuated FPG effect in the balanced analysis. This dissociation implies that the benefits of IER extend beyond gluco-regulation, likely mediated through pleiotropic mechanisms (24, 25). Preclinical and clinical mechanistic studies suggest that cyclical fasting enhances mitochondrial function, reduces oxidative stress, and modulates inflammatory pathways (26, 27). Our subgroup analyses further support this: the pronounced benefit in individuals with higher BMI may reflect improvements in adipose tissue function and systemic insulin sensitivity, while the effect in those with shorter diabetes duration suggests a window of opportunity for beta-cell recovery and metabolic repair (27, 28). The significant medication reduction and high adherence rate (60.12%) further underscore IER’s potential for metabolic remission and practical feasibility (29).

When contextualized within the existing evidence, our findings offer a compelling advance. The Look AHEAD trial established that intensive lifestyle intervention can reduce microvascular complications, but this required sustained weight loss over a decade (30). In contrast, our results suggest that IER was associated with significant clinical benefits within a much shorter timeframe (31). Furthermore, while the ACCORD trial highlighted the limitations of intensive glucose control alone for cardiovascular risk reduction (7), IER appears to dissociate complication risk from glycemia, aligning with a growing understanding that targeting upstream metabolic and inflammatory pathways may be essential for organ protection (32).

The robustness of our primary finding is strengthened by several methodological considerations. The use of IPW to balance numerous baseline covariates, including age, baseline FPG, and insulin use, enhances the validity of our causal inferences. Crucially, the sensitivity analysis restricted to patients without baseline PDDP confirmed the robustness of the intervention effect, demonstrating a 68% lower risk of new-onset PDDP in the IER group. This consistency across analytic approaches, coupled with the generally non-significant interaction effects across most patient subgroups, indicates that the protective effect of IER is generalizable across a broad spectrum of individuals with type 2 diabetes (2). We employed a two-tiered analytical strategy to address the possibility of bias from pre-existing complications: a primary analysis adjusting for baseline PDDP status and a confirmatory sensitivity analysis restricted to patients without baseline PDDP. The concordance of results across these approaches increases confidence in our findings. Several limitations of our study warrant consideration. First, despite the use of advanced causal inference methods (IPW) to balance a comprehensive set of measured covariates, the observational design cannot establish causality, and residual confounding from unmeasured factors remains possible. Potential unmeasured confounders include detailed physical activity patterns, nuanced dietary habits outside the prescribed regimen, socioeconomic status, psychosocial stress, and variations in medication adherence not captured in records. While our sensitivity and subgroup analyses support robustness, the influence of these factors cannot be fully excluded (33). Second, in this retrospective real-world study, baseline differences between cohorts, though addressed with IPW, may introduce residual bias; the control group had a higher baseline risk profile, which, despite statistical adjustment, may still influence the effect size, follow-up BMI data were not consistently available in the clinical records. This is a recognized limitation of real-world data. Future prospective, randomized trials are needed to confirm the efficacy of IER for complication prevention. Such studies should incorporate deeper phenotyping, including biomarkers of oxidative stress, inflammation, and organ function, to elucidate the precise mechanisms involved (34). Extending the follow-up duration will also be critical to determine the long-term sustainability of these benefits and the effect of IER in patients with established advanced complications (35). It is important to note that these interpretations, while biologically plausible, are derived from an observational association, and residual confounding cannot be ruled out.

## Conclusion

This observational study found that IER was associated with a substantially lower risk of Physician-Diagnosed Diabetes Progression, an effect largely independent of short-term glycemic changes. These results suggest structured IER may be a promising non-pharmacological strategy, warranting further randomized trials to confirm causality and define its clinical role.

## Data Availability

Medical data is not suitable for public disclosure. Requests to access these datasets should be directed to Zhiyong Xiao, xiaozy@stu.hunau.edu.cn.
