# Supplementary material for: Intermittent energy restriction and risk of physician-diagnosed diabetes progression: a propensity-weighted real-world cohort study
Source: Front Nutr. 2026 Feb 4;13:1744017. doi: 10.3389/fnut.2026.1744017 (PMC12913150; doi:10.3389/fnut.2026.1744017)
Supplement: Supplementary file 1 [file Table_1.docx]

**Supplementary Content**

eTable 1. Composition of the Human CMNT Diet

eTable 1-1. Caloric Content of the Human CMNT Diet

eTable 1-2. Ingredients of the Human CMNT Diet

eTable 2. Baseline Medication Use in IER Cohort Compared to Control Cohort

eTable 3. Incidence of PDDP in IER Cohort Compared to Control Cohort

eTable 4. Sensitivity Analysis

eTable 4-1. Baseline Characteristics between IER and Control Cohorts without PDDP at baseline

eTable 4-2. Univariate Analysis of Outcomes between ICR and Control Cohorts without PDDP at baseline

eFigure 1. Standardized Mean Differences (SMD) of Covariates Before and After IPW Adjustment between IER and Control Cohorts without PDDP at baseline

eTable 4-3. Outcomes After IPW between IER and Control Cohorts without PDDP at baseline

**eTable 1. Composition of the Human CMNT Diet.**

**eTable 1-1. Caloric Content of the Human CMNT Diet.**

| **Calorie information** | **Solids beverages** | **Fruit and vegetable gruel** | **Composite  nutritional rice** | **Meal replacement  biscuit** |  |
| --- | --- | --- | --- | --- | --- |
|  |  |  |  |  |  |
| Energy density (kcal/100 g) | 576.24 | 533.22 | 358.75 | 489.96 |  |
| Protein (g/100 g) | 7.20 | 3.40 | 10.50 | 7.10 |  |
| Protein % | 5.28 | 2.52 | 11.86 | 5.97 |  |
| Fat (g/100 g) | 50.00 | 30.80 | 1.80 | 18.20 |  |
| Fat % | 84.09 | 52.39 | 4.66 | 35.13 |  |
| Carbohydrates (g/100 g) | 14.50 | 60.80 | 73.90 | 70.00 |  |
| Carbohydrates % | 10.63 | 45.09 | 83.48 | 58.90 |  |
| Fiber (g/100 g) | 23.90 | - | - | 8.20 |  |
| Sodium (mg/100 g) | 63.00 | 95.00 | 41.20 | 264.00 |  |

**eTable 1-2. Ingredients of the Human CMNT Diet.**

| **Diet item** | **Daily intake** | **Ingredients** |  |
| --- | --- | --- | --- |
| **Breakfast** |  | |  |
| Fruit and vegetable gruel | 50 g | Fresh pumpkins, pumpkin seed kernel oil, maltodextrins, isomalto-oligosaccharide, casein, resistant dextrin, sodium ascorbate, potassium citrate, mono- and diglycerides of fatty acids esters, vitamin E, tea polyphenols, and silicon dioxide. |  |
|  |  |  |  |
|  |  |  |  |
|  |  |  |  |
|  |  |  |  |
| **Lunch** |  |  |  |
| Solids beverages | 25 g | Pumpkin seed kernel oil, isomalto-oligosaccharide, casein, resistant dextrin, sodium ascorbate, potassium citrate, mono- and diglycerides of fatty acids esters, vitamin E, tea polyphenols, and silicon dioxide. |  |
|  |  |  |  |
|  |  |  |  |
|  |  |  |  |
| Composite nutritional rice | 50 g | Homologous medicine and food substance: *Fructus lycii*, *Ganoderma lucidum*, Folium Mori, *Poria cocos*, *Dioscorea opposita* Thunb. (Chinese yam), Radix Puerariae, *Cordyceps militaris*, and *Momordica grosvenori*. Wholegrains and others: Rice, millet, corn, buckwheat, quinoa, oat, spinach powders, lily root flour, cucumber powders, mushroom powder, wheat dietary fiber, bitter melon, pumpkins, potato, purple potato, sweet potato, mung bean, konjac flour, inulin, and edible refined salt. |  |
|  |  |  |  |
|  |  |  |  |
|  |  |  |  |
|  |  |  |  |
|  |  |  |  |
|  |  |  |  |
|  |  |  |  |
|  |  |  |  |
| **Dinner** |  |  |  |
| Solids beverages | 25 g | Pumpkin seed kernel oil, isomalto-oligosaccharide, casein, resistant dextrin, sodium ascorbate, potassium citrate, mono- and diglycerides of fatty acids esters, vitamin E, tea polyphenols, and silicon dioxide. |  |
|  |  |  |  |
|  |  |  |  |
|  |  |  |  |
| Meal replacement  biscuit | 30 g | Medicine food homologous plants, wholegrains, and others: *Dioscorea opposita* Thunb. (Chinese yam), wheat flour, MAIKERENJIA, mix powder (quinoa, white kidney, wheat germ, azuki bean, black beans, yellow beans, liriopes radix, glutinous rice, black rice, maize, round bract *Plantago ovata* husk power, oat, buckwheat, hawthorn, roselle, millet, brown rice, Chinese jujube, Chinese wolfberry, pecan nuts, chia seed, black sesame, white sesame, shiitake mushroom, *Laminaria hyperborea*, and coffee), edible vegetable oils, potato protein, wheat dietary fiber powder, resistant dextrin, maltodextrin, and L-arabinose. |  |
|  |  |  |  |
|  |  |  |  |
|  |  |  |  |
|  |  |  |  |
|  |  |  |  |
|  |  |  |  |
|  |  |  |  |
|  |  |  |  |
|  |  |  |  |
|  |  |  |  |

**eTable 2. Baseline Medication Use in IER Cohort Compared to Control Cohort.**

| **Drugs** | **Total number of participants** | **%** | **Total number of participants** | **%** |
| --- | --- | --- | --- | --- |
|  | **IER cohort** |  | **Control cohort** |  |
| Overall | 1,069 | 100% | 1,099 | 100% |
| Taking metformin | 597 | 55.85% | 408 | 37.12% |
| Taking SGLT2 inhibitors | 97 | 9.07% | 136 | 12.37% |
| Taking DPP-4 inhibitors | 47 | 4.40% | 44 | 4.00% |
| Taking GLP-1 analogs | 5 | 0.47% | 8 | 0.73% |
| Taking sulfonylureas | 228 | 21.33% | 180 | 16.38% |
| Taking pioglitazone | 29 | 2.71% | 14 | 1.27% |
| Taking meglitinides | 32 | 2.99% | 20 | 1.82% |
| Taking acarbose | 156 | 14.59% | 123 | 11.19% |
| Taking insulin | 156 | 14.59% | 451 | 41.04% |

This table presents the types of antidiabetic medications used by participants in both cohorts at baseline, highlighting significant differences in drug use between the cohorts.

**eTable 3. Incidence of PDDP in IER Cohort Compared to Control Cohort.**

| **PDDP** | **Total number of participants** | **Total number of participants** |
| --- | --- | --- |
|  | **IER cohort** | **Control cohort** |
| Overall | 19 | 109 |
| Diabetic nephropathy | 4 | 36 |
| Diabetic retinopathy | 7 | 20 |
| Diabetic neuropathy | 2 | 13 |
| Peripheral arterial disease (PAD) | 0 | 9 |
| Diabetic nephropathy and diabetic retinopathy | 3 | 8 |
| Diabetic ketoacidosis (DKA) | 0 | 4 |
| Diabetic nephropathy and diabetic neuropathy | 1 | 4 |
| Diabetic neuropathy and diabetic retinopathy | 2 | 3 |
| Diabetic retinopathy and PAD | 0 | 3 |
| Diabetic foot | 0 | 3 |
| Cardiovascular disease | 0 | 2 |
| Hyperosmolar hyperglycemic state | 0 | 1 |
| Diabetic neuropathy and PAD | 0 | 1 |
| Diabetic nephropathy and DKA | 0 | 1 |
| Gastroparesis | 0 | 1 |

This table compares the incidence of PDDP between the IER cohort and the control cohort. Data are presented as the absolute number of new cases.

**eTable 4. Sensitivity Analysis**

**eTable 4-1. Baseline Characteristics between IER and Control Cohorts** **without PDDP at baseline**

| Variables | Total (n = 1788) | ICR (n = 1069) | Control (n = 719) | *p*-value |
| --- | --- | --- | --- | --- |
| Sex |  |  |  | 0.093 |
| Male | 1133 (63) | 689 (64) | 444 (62) |  |
| Female | 655 (37) | 380 (36) | 275 (38) |  |
| Origin | 14 (12, 16) | 15 (6, 25) | 14 (14, 14) | 0.465 |
| Follow-up duration (days) | 166.06 (122, 232) | 152.21 (119.89, 200.03) | 198 (128, 294.5) | < 0.001 |
| FBG before intervention | 8.5 (6.9, 11.6) | 7.8 (6.51, 9.8) | 10.63 (7.62, 14.8) | < 0.001 |
| PDDP of diabetes at Baseline |  |  |  | 1 |
| 0 | 1788 (100) | 1069 (100) | 719 (100) |  |
| Insulin use at baseline |  |  |  | < 0.001 |
| Untreated | 1389 (78) | 913 (85) | 476 (66) |  |
| Treated | 399 (22) | 156 (15) | 243 (34) |  |
| Age (years) | 57 (51, 63) | 55 (50, 60) | 61 (54.5, 68) | < 0.001 |
| BMI (kg/m2) | 23.66 (21.71, 25.56) | 23.53 (21.8, 25.35) | 23.88 (21.48, 25.95) | 0.285 |
| Antidiabetic drugs at baseline |  |  |  | < 0.001 |
| 0 | 303 (17) | 248 (23) | 55 (8) |  |
| 1 | 728 (41) | 360 (34) | 368 (51) |  |
| 2 | 557 (31) | 339 (32) | 218 (30) |  |
| 3 | 166 (9) | 98 (9) | 68 (9) |  |
| 4 | 34 (2) | 24 (2) | 10 (1) |  |
| Duration of diabetes (years) | 5 (1, 10) | 5 (2, 10) | 5 (1, 10) | 0.052 |

This table presents the baseline demographic and clinical characteristics of the IER and control cohorts, including age, fasting Plasma glucose (FPG), BMI, duration of diabetes, insulin use, and antidiabetic drug use. Significant differences between cohorts are marked with *p* < 0.05.

**eTable 4-2. Univariate Analysis of Outcomes between ICR and Control Cohorts without PDDP at baseline**

| **Variables** | **Total**  **(n = 2168)** | **ICR**  **(n = 1069)** | **Control**  **(n = 719)** | ***p*-value** |
| --- | --- | --- | --- | --- |
| PDDP |  |  |  | < 0.001 |
| 0 | 1411 (79) | 1050 (98) | 361 (50) |  |
| 1 | 371 (21) | 13 (1) | 358 (50) |  |
| 2 | 6 (0) | 6 (1) | 0 (0) |  |
| FBG change | -1.4 (-3.6, 0.2) | -1.4 (-3.1, -0.1) | -1.46 (-5.36, 1.05) | 0.654 |
| Drug reduction |  |  |  | < 0.001 |
| Reduce | 990 (55) | 418 (39) | 572 (80) |  |
| No reduction | 798 (45) | 651 (61) | 147 (20) |  |

Univariate analysis comparing FBG changes, and drugs reduction between ICR and control cohorts. *p*-values below 0.05 indicate statistically significant differences between the cohorts.

**eFigure 1. Standardized Mean Differences (SMD) of Covariates Before and After IPW Adjustment between IER and Control Cohorts without PDDP at baseline**

**
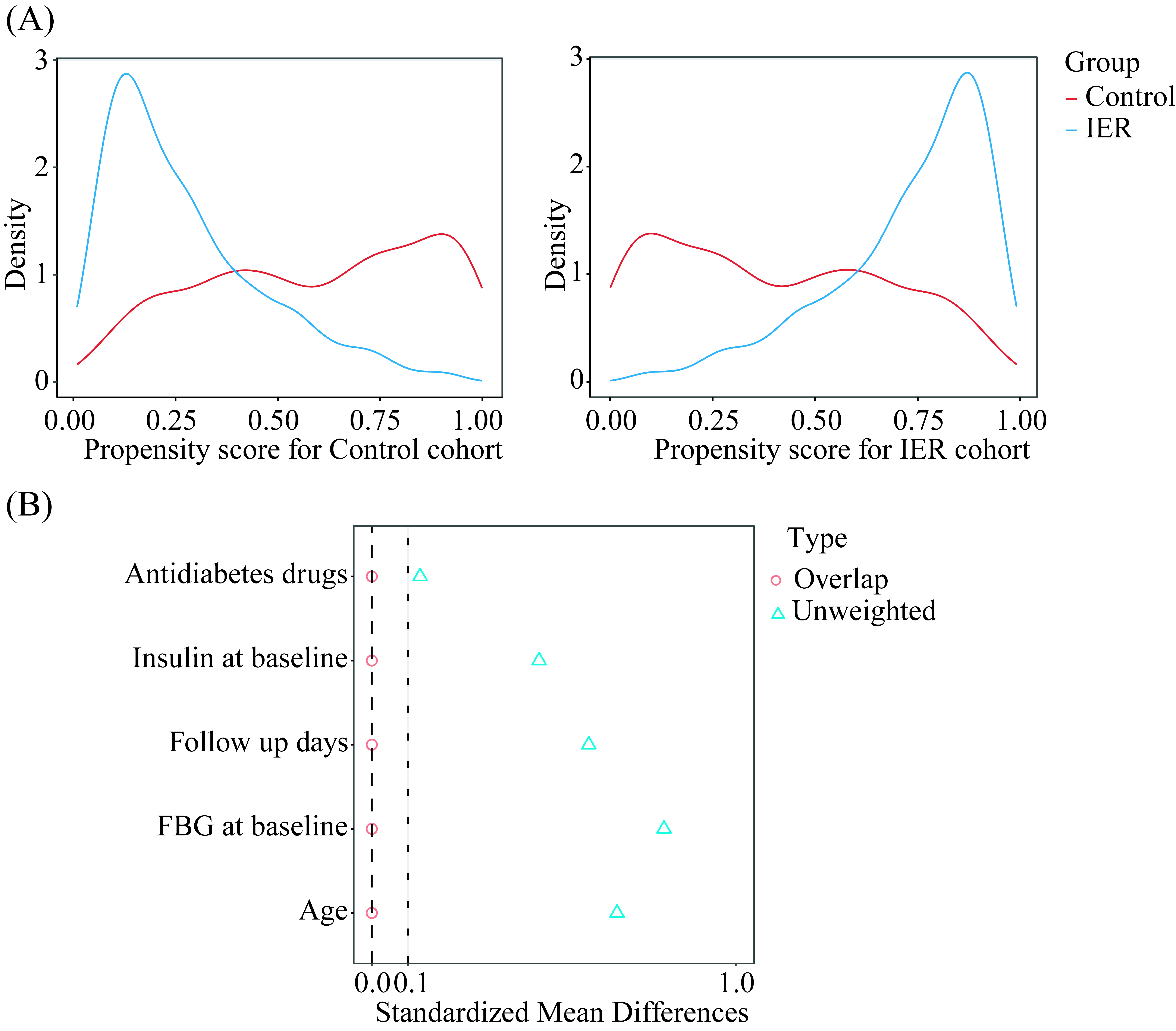
**

(A) Density plots of propensity scores for the intervention and control cohorts before (left) and after (right) matching. The red solid line represents the IER cohort, while the blue dashed line indicates the control cohort. Propensity score matching enhanced the alignment of score distribution between the two groups. (B) SMD for covariates before (△) and after (○) propensity score matching. Vertical dashed lines indicate the threshold of 0.1. Post-matching SMD values are closer to zero, demonstrating that matching effectively reduced covariate imbalances and improved comparability between cohorts. .

**eTable 4-3. Outcomes After IPW between IER and Control Cohorts without PDDP at baseline**

| **Variables** | **Estimate (95% CI)** | **Std. error** | ***p*-value** |
| --- | --- | --- | --- |
| PDDP | 0.05 (0.04–0.08) | 0.01 | < 0.001 |
| Drug reduction | 5.34 (4.76 to 6.00) | 0.32 | < 0.001 |
| FBG change | 0.37 (-0.05 to 0.79) | 0.21 | 0.081 |

This table presents reduction in medication use, changes in FPG, and incidence of PDDP after adjustment for confounders using IPW. Estimates and 95% CIs are provided, with statistical significance indicated by *p* < 0.05.
